# Bergman vs. Volker

**Published:** 1876-11

**Authors:** 


### Editorial.

### Bergman vs. Volker.

We publish elsewhere a communication from Dr. Charles C. F. Gay, in reference to our report of the trial of the case of Bergman vs. Volker. As nothing was further from our motive in publishing the report than a desire to misrepresent Dr. Gay in any manner, we are very happy to be able to give place in this issue to his rejoinder; we must, however, assume the right of correcting or modifying some of his statements. In regard to the alleged error on page 92, the manuscript, still in our possession, reads: "Dr. White's (measurement) varied from 2¾ of an inch to 2 inches," and Dr. White in his testimony, which we did not give in full, swore that his first measurement made 2¾ of an inch shortening; that his second was 2 inches. In reference to the word eczematous, it occurs at the opening of Dr. Gay's testimony on page 90, and not on 92. Its bearing on the question at issue we cannot see, we are, however, willing to call it "scales." If the "several" errors to which Dr. Gay alludes are of like importance, we are not surprised that he did not enumerate them. The first two lines on page 93 are exactly as they read in the reporter's manuscript before us, and if not the exact words carry out the idea which we heard Dr. Gay express on the stand at this very point in the course of his testimony. To prevent further error, Dr. Gay has in the present communication, corrected his own proof. In regard to the errors in the testimony of the other witnesses, we know of none except in our report of Dr. Rochester's. On page 98 the types make him say that shortening of from two to *three* and one-half inches was found. This error we were about to correct in another manner, at Dr. Rochester's request, but take this occasion to do so. It should read from two to *two* and one-half inches. In regard to our Editorial remarks, we are alone responsible for them; neither did we consult our imagination, nor call in the aid of spirits, either rectified, good, bad, or indifferent. We deny all dealing with such unstable confreres. The basis of our remarks upon Dr. Gay's "very grave charge" is this: In the *Sunday News* of Oct. 20th, may be found an interview had by one of the representatives of that paper with Dr. Gay, in the course of which he twice takes occasion to charge the experts appointed by the court, with having "combined" to break down his diagnosis. These gentlemen were appointed to inform the court of Bergman's condition, to have "combined" either to uphold or break down the diagnosis made by Dr. Gay, unless the facts sustained them, would have been committing perjury.

The very natural inference to be drawn from Dr. Gay's rather ill-advised remarks during the whole of this published interview is, that he considered the combination made to break him down, no matter what the facts of the case were. He denies to us the privilege (which we do not consider one) of doing exactly what he has done, and in the public prints. We do esteem it a privilege in putting on record his denial of an affront, at once, so grave and uncalled for.

In his recapitulation of the points of the case, we are sure Dr. Gay would not have his readers infer, as they might from his use of quotations, etc., that the patient continued under Dr. Miner's care from Dec. 11, '75, until Feb., '76, when another physician "commenced" treatment. Dr. Miner saw him but once, as our readers are aware from his testimony, and then as a consultant, on account of the dropsy.

We are very positive that he is mistaken in regard to the measurements made by Dr. White, and, we therefore again call his attention to the fact, which the notes of the stenographer will coroborate, that Dr. White's measurements, as sworn to by himself, made the shortening from 2¾ to 2 inches, so that the differences between the first and Dr. Miner's last is ⅛ instead of 1⅞ inches. The statement made that Bergman was confined to his bed nine months, Dr. Gay will find to be incorrect by referring to the testimony. Some of the physicians under whose care he came, state distinctly that he could not lie down, so great was the "œdema." Dr. Miner's testimony, we are sure he is ready to stand by, but without consultation with him, we will simply say in explanation of Dr. Gay's remarks upon it, that what he said in reference to the union of intra-capsular fracture was in answer to the question, "does not this fracture sometimes unite by bone?" or a question of that import. The remark that it united in nearly its old position, was in reply to testimony already before the court, in which an attempt was made to show that to obtain two inches shortening, the head would have to be carried down upon the shaft two inches.

Following out the plan which we adopted at the commencement of this article, of taking up such points as we deemed demanding notice, in the order in which they occurred in the paper, we come to the last thing in the paper concerning which we care at this time to make any remarks. Dr. Gay objects to our assertion that he candidly said that he did not reduce the dislocation. After reading his denial of making any such candid statement we must confess that we were probably mistaken, for from this point, to the end of the article, we are at a loss to determine whether he thinks the head of the bone now in the acetabulum or in the ischiatic notch. We were inclined to think when we heard him swear that he thought the head of the bone in the ischiatic notch, that such a statement was a pretty candid avowal that it was not in the acetabulum, or in other words, that the dislocation was not *reduced*, but on reading his reply, we cannot find any definite and exact statement as to the position of the head of the bone. To say that he has "reduced" the head of a femur from the dorsum ilii into the ischiatic notch is to lay claim to a "reduction" with which we are not familiar, although tolerably informed in the literature of the subject. With such a use of the word "reduced" of course we were out of the way in our statement.

With the surgical questions involved, we have in this connection nothing to do, we would respectfully recommend their reference to a High "Joint" Commission. While we have our own opinion in regard to the matter, we do not care to enter into a discussion of the points, nor can we allow a further consideration of them, in a controversial manner, in these columns.

